# Sanitary Legislation

**Published:** 1858-10

**Authors:** 


### SANITARY LEGISLATION.

During the last session, three Public Health measures have passed into law in England. The first, *An Act to alter and amend the Metropolis Local Management Act* (1855), *and to extend the powers of the Metropolitan Board of Works for the purification of the Thames and the Main Drainage of the Metropolis,* leaves the whole question and action of purifying and draining with the Metropolitan Board. The second, entitled *The Local Government Act,* 1858, is an Act to amend the Public Health Act of 1848, and to allow an extension of the Act of 1848 to the government of towns and populous districts. This Act may be adopted,

1. In corporate boroughs to which the Public Health Act, 1848, has not been applied, by a resolution of the Council assembled at a meeting held for the purpose : provided always, that this Act shall not be adopted in corporate boroughs until after the election of councillors, on the first day of November, one thousand eight hundred and fifty-eight :

2. In other places under the jurisdiction of a Board of Improvement Commissioners, where all or part of the Commissioners are elected by ratepayers, or by owners and ratepayers, by a resolution of such Improvement Commissioners assembled at a meeting held for the purpose :

3. In all other places having a known or defined boundary, by a resolution of the owners and ratepayers :

But no such resolution, passed by any Council or Board of Improvement Commissioners, shall be valid unless a month's previous notice of the meeting, and of the purpose thereof, has been given in manner in which notices of meetings of such Council or Board of Commissioners are usually given, nor unless two-thirds of the members present at the meeting concur in the resolution for such adoption ; and it shall be lawful for the chairman of any such meeting, with the consent of a majority of the members present, to adjourn the same from day to day.

The third Act, entitled *The Public Health Act,* 1858, is an Act for vesting in the Privy Council certain powers for the protection of the public health : it transfers the powers previously invested in the General Board of Health, under the "Diseases Prevention Act, 1855", to the Privy Council. The Act is only to be in force until the first day of August 1859.

